# COVID-19 and the Impact on the Cranio-Oro-Facial Trauma Care in Italy: An Epidemiological Retrospective Cohort Study

**DOI:** 10.3390/ijerph18137066

**Published:** 2021-07-01

**Authors:** Fausto Famà, Roberto Lo Giudice, Gaetano Di Vita, João Paulo Mendes Tribst, Giorgio Lo Giudice, Alessandro Sindoni

**Affiliations:** 1 Department of Human Pathology in Adulthood and Childhood “G. Barresi”, University Hospital “G. Martino” of Messina, Via Consolare Valeria 1, 98123 Messina, Italy; fausto.fama@unime.it; 2 Department of Surgical Oncological and Stomatological Sciences, University of Palermo, Piazza Marina 61, 90133 Palermo, Italy; gaetano.divita@unipa.it; 3 Department of Dentistry, University of Tautabé (UNITAU), Taubaté 12030-040, Brazil; joao.tribst@unesp.br; 4 Maxillofacial Surgery Unit, Multidisciplinary Department of Medical-Surgical and Dental Specialities, University of Campania “Luigi Vanvitelli”, Viale Abramo Lincoln 5, 80138 Naples, Italy; giorgio.logiudice@gmail.com; 5 Department of Public Health and Infectious Diseases, Sapienza University of Rome, Piazzale Aldo Moro 5, 00185 Rome, Italy; alessandrosindoni@alice.it; 6 Direzione Sanitaria, Azienda Ospedaliero-Universitaria Policlinico Umberto I, 00161 Rome, Italy

**Keywords:** COVID-19, cranio-oro-facial traumatology, retrospective cohort study

## Abstract

The coronavirus disease 2019 (COVID-19) has deeply modified the organization of hospitals, health care centers, and the patient’s behavior. The aim of this epidemiological retrospective cohort study is to evaluate if and how the COVID-19 pandemic has determined a modification in cranio-oro-facial traumatology service. Methods: The dataset included hospital emergency room access of a six-month pre-pandemic period and six months into pandemic outbreak. The variables collected were: patient age, gender, type of emergency access with relative color code, Glasgow Coma Scale Score, type of discharge. Results: 537 vs. 237 (pre-pandemic vs. pandemic) patients accessed the hospital emergency room and the mean age decreased from 60.79 ± 25.34 to 56.75 ± 24.50 year. Yellow and green code access went from 28.9% and 66.1% to 37.5% and 57.7% (pre-pandemic vs. pandemic). Glasgow Coma Scale (GCS) shows an increase of 16.6% vs. 27.8% of 15 grade score, a 28.7% vs. 28.5% of the 14 grade score and reduction of 13 and 12 grade 40.2% and 14.5% vs. 37.1 and 9.7% (pre-pandemic vs. pandemic). Conclusions: Since the COVID-19 outbreak continues, epidemiological data are still necessary to perform public health intervention strategies and to appropriately predict the population needs, in order to properly manage the COVID-19 related to oral pathologies as well as the most common health problems.

## 1. Introduction

The Coronavirus disease 2019 (COVID-19) was declared as a pandemic on 11 March 2020 by the World Health Organization (WHO) [1]. In Italy, COVID-19 was firstly detected in February 2020, and a Task Force and a special commission was organized to properly manage the outbreak [2]. On March 9, a National Lockdown was declared after a continuous increase in death and contagion cases that up to date (27 April 2021) count 3,971,114 total cases, 3,398,763 dismissed from hospital/healed, and 119,539 deaths [3]. The route of transmission may vary but the infection spread mainly through coughing, sneezing, and saliva [4]. The most common symptoms include body temperatures >37.4 °C, dry cough, dyspnea, asthenia, muscle pain, headache, sore throat, diarrhea, and vomiting [5].

COVID-19 has deeply modified the organization of hospitals and health care centers that have been called to a redistribution of workforce and resources to respond to a changed public health necessity. In particular, to maximize the hospital beds and the resources available, a reduction in elective surgeries and a deep rescheduling of all non-life-treat intervention were carried out according to new specific guidelines in each medical branch [6,7,8].

The occurrence of a cranio-oro-facial trauma can negatively affect patients’ health generating social, economic, and professional issues [9,10]. Many factors such as risky sports, altered overjet of the upper central incisors, and age-related lifestyle, can be associated to an event that can lead to a cranio-oro-facial trauma [9,10]. The severity of this kind of trauma and the complexity of the anatomical district involved, can require a multidisciplinary approach [9]. However, in the pandemic period, the most common health treatments can become a challenge for the professionals that are trying to offer the best treatment and to avoid COVID-19 infection [11,12].

A previous report that evaluated if underlying diseases such as diabetic mellitus and hypertension are risk factors for the severity and mortality of COVID-19 patients showed an increased hospitalization duration and mortality in patients with confirmed COVID-19 [13]. In addition, underlying diseases such as diabetes, hypertension, cancer, chronic kidney disease, heart failure, and mental disorders increased COVID-19 mortality [13]. According to the authors, there is a need to develop more elaborate treatment guidelines and strategies in order to improve the prognosis of COVID-19 patients [13]. In these circumstances, it can be even more complex to properly manage traumatic injuries, which can result in longer hospitalization duration.

As the COVID-19 outbreak continues, epidemiological data are still necessary to guide awareness and public health intervention strategies.

Our epidemiological retrospective cohort study aimed at evaluating if and how the COVID-19 pandemic has determined a modification in cranio-oro-maxillo-facial traumatology service, in terms of number and type of hospitalizations, accesses, accidents, and emergency attendances.

For this purpose, we compared data related to the hospital emergency room access of a six-month pre-pandemic period (1 March 2019–1 August 2019) with a six-month period into the pandemic outbreak (1 March 2020–1 August 2020).

## 2. Materials and Methods

This epidemiological retrospective cohort study was carried out in compliance with the principles outlined in the Declaration of Helsinki.

The research followed the STrengthening the Reporting of OBservational studies in Epidemiology (STROBE) checklist available upon request to the corresponding author.

The present study was conducted in Messina, a city with a population of 225,879, evaluating the patients that referred to the emergency room of Policlinico Universitario G. Martino [14]. The hospital is within the Italian National Health Care System.

Data retrieved from the digital archive of access and diagnosis of the hospital emergency room were collected and evaluated in two different periods: a pre-pandemic (1 March 2019–1 August 2019) and a pandemic one (1 March 2020–1 August 2020).

Inclusion criteria were the following: presenting to emergency services, diagnosis of brain traumatic injury, and/or cranio-oro-facial trauma.

The diagnosis followed the guidelines of American College of Surgeon, Trauma Quality Improvement Program: best practices in the management of traumatic brain injury (January 2015), adapted to the hospital organization. [15]

We collected the following data: patient age, gender, type of emergency access with relative code, Glasgow Coma Score, patient discharge status. Codes of access were as follows: red code—very critical, life-threatening, top priority, immediate access to treatment; yellow code—on average critical, presence of evolutionary risk, potential danger of life, performance that cannot be deferred; green code—not very critical, absence of evolutionary risks, deferrable services. Types of patients discharge were: refusal, home, ambulatory consultation, hospitalization.

### Statistical Analysis

Continuous variables were expressed as a mean with standard deviation and discrete quantitative data as absolute frequencies. Student’s *t*-test was used to analyze the differences between age means. Comparisons in the percentages of the code of access between the pandemic and the pre-pandemic group were performed with the chi-square test. Glasgow Coma Score values were detailed in percentages and their differences were analyzed by the chi-square test. The chi-square test was also used to assess differences in percentages of type of discharge. The null hypothesis is that the pandemic does not affect the emergency services and the patient’s profile. Values of *p* < 0.05 were considered statistically significant.

## 3. Results

### 3.1. Demographic Characteristics

Patients in the pre-pandemic group were 537, being 324 men and 213 women, whereas patients in the pandemic group were 267, being 146 men and 121 women: the difference did not reach statistical significance (χ^2^ = 2.347; df = 1; *p* = 0.126). The mean age in the pre-pandemic group was 60.79 ± 25.34 years, while the mean age in the pandemic group was 56.75 ± 24.50 years. The lower age found in people referred in the pandemic period with respect to the age in the pre-pandemic group reached statistical significance (*p* = 0.031).

### 3.2. Code of Access

**Figure 1:**
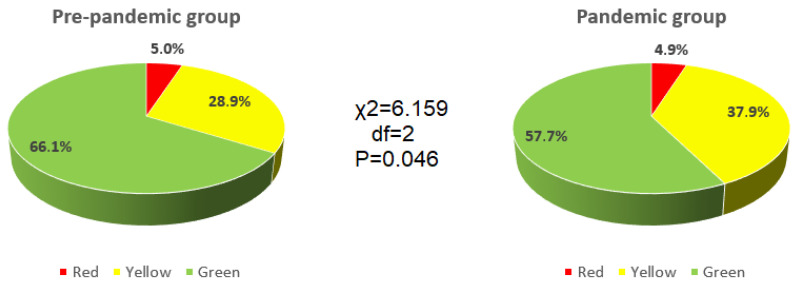
Code of access: pre-pandemic vs. pandemic group comparison.

In the pre-pandemic group, 27/537 (5.0%) accessed the emergency department with a red code, 155/537 (28.9%) with a yellow code and 355/537 (66.1%) with a green code. In the pandemic group, 13/267 (4.9%) accessed the emergency department with a red code, 100/267 (37.5%) with a yellow code and 154/267 (57.7%) with a green code. The higher percentage of yellow codes and the lower percentage of green codes in the pandemic group with respect to the pre-pandemic group reached statistical significance (χ^2^ = 6.159; df = 2; *p* = 0.046) (Figure 1).

### 3.3. Glasgow Coma Score

Glasgow Coma Score values in the pre-pandemic group were: 15 (*n* = 89, 16.6%), 14 (*n* = 154, 28.7%), 13 (*n* = 216, 40.2%), 12 (*n* = 78, 14.5%). Glasgow Coma Scale values in the pandemic group were: 15 (*n* = 66, 27.8%), 14 (*n* = 76, 28.5%), 13 (*n* = 99, 37.1%), 12 (*n* = 26, 9.7%). Differences between percentages were statistically significant (χ^2^ = 9.570; df = 3; *p* = 0.021), since in the pandemic group was found a higher percentage of score 15 and a lower percentage of score 12 with respect to the pre-pandemic group.

### 3.4. Type of Discharge

Types of discharge in the pre-pandemic group were: refusal (*n* = 129, 24.0%), home (*n* = 214, 39.9%), ambulatory consultation (*n* = 122, 22.7%), and hospitalization (*n* = 72, 13.4%). Types of discharge in the pandemic group were: refusal (*n* = 80, 30.0%), home (*n* = 111, 41.6%), ambulatory consultation (*n* = 34, 12.7%), and hospitalization (*n* = 42, 15.7%). Differences between percentages in refusal, ambulatory consultation, and hospitalization seen in the pandemic group with respect to the pre-pandemic group were statistically significant (χ^2^ = 12.393; df = 3; *p* = 0.006).

**Figure 2:**
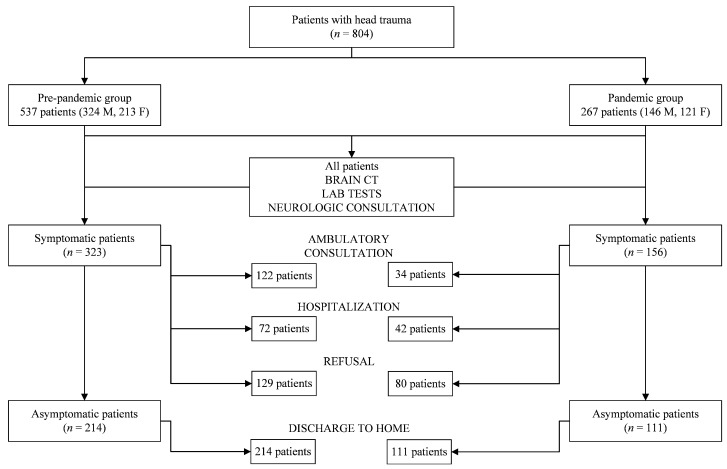
Diagnostic breakdown flowchart.

The diagnostic breakdown along with an overview of the sample analyzed is summarized in Figure 2.

## 4. Discussion

The COVID-19 pandemic has determined deep modifications in public health services, with many epidemiological retrospective cohort studies that evaluated data for different medical branches [16,17].

During this pandemic period, the limited resources in terms of active medical force and hospitalization beds in the Intensive Care Units require a careful planning of the patient’s needs in a strict coordination with the hospital’s needs; therefore, cohort data are a valuable asset to perform a strategic plan of the hospital health providing capability.

Our dataset compared a pre-pandemic six-month period with a six-month pandemic period (1 March 2019–1 August 2019 vs. 1 March 2020–1 August 2020) in the same hospital using the same diagnostic criteria, to show if any modification for the evaluated parameters (age, gender, type of emergency access, Glasgow Coma Score, type of discharge) occurred. Therefore, observing the results of this study, it was possible to suggest that the pandemic had an impact on emergency services and patients’ profiles.

Hospital avoidance, due to fear of infection, has been documented in literature, and data suggest a reduction of 35.2% in term of overall hospital accesses [18]. The data in this study, moreover, highlight that the avoidance increased up to 50% in case of cranio-oro-facial traumatology service comparing the pre-pandemic vs. pandemic period (537 vs. 237). Despite the non-statistical significance of this comparison in term of raw data of access (*p* = 0.126), statistical significance was found when comparing patients’ age (mean, 60.79 ± 25.34 vs. 56.75 ± 24.50) and color access codes to the emergency department between the two groups.

In agreement with the results of this study, a retrospective multicenter comparative study in France also hypothesized that an increased number of patients might have refused elective procedures out of fear of contracting SARS-CoV-2 during their hospitalization [19].

In particular, the significant reduction in the incidence of maxillofacial trauma requiring surgical management should be taken into account in order to reallocate resources more accurately to improve the management of other pathologies for which treatment cannot be postponed without affecting the patient’s vital prognosis [18]. This consideration can also be applied to the present study.

The results of this study are in line with the literature that underlines how emergency department visits among the adult and pediatric population showed a large decrease in sports-related and motor vehicle-related injuries. [20,21]

According to the American Dental Association, dental emergencies could be potentially life-threatening and could require immediate treatment to alleviate severe pain and infection and to stop ongoing tissue bleeding [22]. Considering the present study, also a previous retrospective investigation in Milan suggests that the COVID-19 pandemic has highly affected dental activities in Italy [22]. The authors found that the number of patients was inversely associated with the COVID-19 pandemic evolution with significantly less patients accessing hospital emergency rooms for urgent dental care during lockdown.

Cranio-oro-facial traumatology service, in terms of hospitalizations, accidents, and emergency attendances has a wide spectrum of patients, divided also, according to the lesion severity, into a color code scale ranging from white/green (no urgency or not very critical situation) to red code with life-threatening lesions for patients that need to have immediate access to treatment and require hospitalization. Our data suggested a non-substantial modification in red code access (5% vs. 4.9%) but a statistically significative modification in yellow and green code access that goes from 28.9% and 66.1% in the pre-pandemic period to 37.5% and 57.7% during the pandemic period (*p* = 0.046). These data could be compared with those of a previous paper that evaluated the impact on patient healthcare management in oral and maxillofacial surgery (OMFS) in Germany and found a reduction in ward capacities and number of surgical procedures ranging from 17 to 78% [23].

Patients were screened using the Glasgow Coma Scale (GCS). This scale, developed by Taesdale and Jennet in 1974, is used to evaluate a person’s level of consciousness after a brain injury and allows evaluating the level of consciousness, identifying neurologic dysfunction, predicting prognosis, and having a standardized communication among health professionals. It is used worldwide to follow up patients’ progress and to perform an objective evaluation of their level of consciousness. The evaluation is based on a score ranging from 3 to 15, divided in three assessment categories: eye opening, verbal response, and motor response. These parameters could be evaluated trough observation of spontaneous activities and use of verbal and/or painful stimuli [24].

The analysis of Glasgow Coma Scale scores resulted in increased access of a grade 15 GCS score, a non-modification of grade 14, and a slight reduction of grades 13 and 12.

This data could be related to the modification of the kind of traumas and their modified nature linked to the deep modification of the lifestyle during the pandemic [20,21].

In consideration of all the data evaluated in our analysis, the null hypothesis has been rejected.

In the pandemic period, with a reduction of the available hospitalization beds, these data could be useful to predict the need for non-deferrable hospitalizations for this medical branch, and to manage hospital organization to respond to the evident reduction in elective surgery, reported in the majority of countries, versus emergency procedures [24].

Medical literature shows how the pandemic outbreak has also modified the patients discharge from hospital preference, probably due to fear for possible SARs-COV-2 contact in the hospital [25,26]. Our data suggest how both treatment refusal and home treatment increased, while ambulatory consultation decreased. In addition, the hospitalization increased reaching statistical significance. These findings underline how the fear for this infection has modified patients’ behaviors, resulting in a different logistic necessity from the hospital [26].

According to the literature, the COVID-19 pandemic has modified the spectrum of medical procedures performed also in other countries [11]. The emergency and invasive surgical procedures has significantly increased, while planned elective procedures have decreased. In subsequent months, the reduced number of patients was compensated by a higher number of procedures performed per visit [11]. The present study showed a similar pattern for the patients with yellow and green code, without difference for the red code cases before and during the pandemic. This is an expected result if we consider that the patients with reduced risk of permanent injury could not be attended in the hospital.

Despite that COVID-19 pandemic impacted routine healthcare, the management of traumatic injuries has remained a priority in patient-care service of oral and maxillofacial practice [26,27]. A previous report showed a significant increase in oral and maxillofacial injuries associated with falls at home during the lockdown scenario in Sri Lanka, reporting that further investigation should be carried out in order to prevent these traumatic injuries [27]. Accordingly, the present study supports this finding, showing that the patient’s profile have been modified during the pandemic period, and this data should be considered as part of the logistic and treatment arrangement in health centers.

Some authors applied a mathematical approach to assist the surgical list reduction evaluating the results from a managerial point of view [28]. They affirmed that it is important to consider the patients prioritization by the hospital authorities, following the institution’s clinical, ethical, and administrative procedures. Such procedures could include: the inevitable revision of the prioritization criteria; changes in the protocols as well as the waiting list management criteria; or a pronounced change in the demand for surgical procedures limitations of clinical resources [28]. In this sense, the present study provided an important clinical data, which could be evaluated and considered during the cranio-oro-facial trauma care service organization.

A systematic review described the COVID-19 as an infectious disease that primarily affects the lungs. Its multi-organ involvement is responsible for several prolonged symptoms, including oral implications [29,30]. In recovered patients, the prolonged symptoms can delay the elective treatments. These prolonged post-COVID-19 symptoms can affect also the post-treatment recovery, including the treatment of cranio-oro-facial traumas; therefore, it should be considered as part of the treatment plan. Even in controlled situations, doctors should consider the scenarios of possible surgical and post-surgical complications, focusing on the preventive actions, and minimally invasive techniques [10].

Thanks to health indicators, it is possible to study the evolution of many phenomena and make valid comparisons. Health indicators can be used to design, evaluate, and refine public health policies [31]. Moreover, these indicators let medical and administrative personnel evaluate the changing reality of each territory and, therefore, to choose with precision the logistic and medical phase to implement, for example, the different phases of de-escalation of the containment measures aimed at stopping the spread of the Sars-CoV-2 virus [32]. For that reason, studies like the present one should be performed to elucidate the pandemic effect on the health services and patients’ profile.

The strength of our study relies on the accurate analysis of the data related to the hospital emergency room accesses of a six-month pre-pandemic period (1 March 2019–1 August 2019) with a six-month period into the pandemic outbreak (1 March 2020–1 August 2020). Our dataset included all patients affected by traumatic brain injury and/or craniofacial trauma: this is a wide spectrum of potential diagnoses, and the analysis of the precise diagnoses is not within the aim of the study. This aspect may apparently represent a study limitation, but the diagnostic breakdown between pre-pandemic and pandemic group is comparable as it follows the same diagnostic guidelines, therefore, it does not skew the results of the study.

Healthcare services took on a completely new dimension due to the COVID-19 pandemic, especially with the development of telehealth services [31], which often represented the only possible form of care provision to the patients with controlled diseases or/and that needs a follow-up. The COVID-19 pandemic has substantially improved the implementation of remote healthcare in healthcare institutions and made it an essential tool for providing healthcare services. Taking into account the increasingly competitive healthcare market, the institutions should focus on achieving high or perfect patient satisfaction ratings to improve the quality of the provided services [32]. However, to provide the adequate telehealth services, the health professionals should be able to adapt to the changed work environment and should be productive and thrive in general [33,34]. Operative strategies like these could be useful to provide the first assistance for green code patients and optimize the hospital receptive capabilities, until the global vaccine campaign achieves a significant level of prevention that will certainly mitigate some of the current challenges that we are facing [35].

## 5. Conclusions

In the specific period and region, COVID-19 has deeply impacted on the healthcare services, also in term of clinical priorities and logistic possibilities.

The results of this study show an avoidance-increase up to 50%, a significant variation in yellow and green code accesses, and a change in GCS scores presentation due to an increase of grade 15 scores, a non-modification of the grade 14, and a slight reduction of grades 13 and 12, confirming the hypotheses in medical literature and underlining the necessity to rearrange organization of hospital emergency and elective services.

Data are mandatory to evaluate the outbreak progression and to perform a correct prediction of the population needs, in order to properly manage COVID-19, its related diseases, and the most common health problems such as the cranio-oro-facial traumas.

## Data Availability

Data available on request from the corresponding author.
